# Examining Sporadic Cancer Mutations Uncovers a Set of Genes Involved in Mitochondrial Maintenance

**DOI:** 10.3390/genes14051009

**Published:** 2023-04-29

**Authors:** Armando Moreno, Allison Taffet, Elissa Tjahjono, Quinton L. Anderson, Natalia V. Kirienko

**Affiliations:** Department of BioSciences, Rice University, 6100 Main St, MS140, Houston, TX 77005, USA; am161@rice.edu (A.M.); allison.taffet@nyulangone.org (A.T.); et19@rice.edu (E.T.); ander1214@gmail.com (Q.L.A.)

**Keywords:** mitochondria, *Caenorhabditis elegans*, cancer, mitophagy, neurodegeneration, development, bioinformatics, *pps-1*

## Abstract

Mitochondria are key organelles for cellular health and metabolism and the activation of programmed cell death processes. Although pathways for regulating and re-establishing mitochondrial homeostasis have been identified over the past twenty years, the consequences of disrupting genes that regulate other cellular processes, such as division and proliferation, on affecting mitochondrial function remain unclear. In this study, we leveraged insights about increased sensitivity to mitochondrial damage in certain cancers, or genes that are frequently mutated in multiple cancer types, to compile a list of candidates for study. RNAi was used to disrupt orthologous genes in the model organism *Caenorhabditis elegans*, and a series of assays were used to evaluate these genes’ importance for mitochondrial health. Iterative screening of ~1000 genes yielded a set of 139 genes predicted to play roles in mitochondrial maintenance or function. Bioinformatic analyses indicated that these genes are statistically interrelated. Functional validation of a sample of genes from this set indicated that disruption of each gene caused at least one phenotype consistent with mitochondrial dysfunction, including increased fragmentation of the mitochondrial network, abnormal steady-state levels of NADH or ROS, or altered oxygen consumption. Interestingly, RNAi-mediated knockdown of these genes often also exacerbated α-synuclein aggregation in a *C. elegans* model of Parkinson’s disease. Additionally, human orthologs of the gene set showed enrichment for roles in human disorders. This gene set provides a foundation for identifying new mechanisms that support mitochondrial and cellular homeostasis.

## 1. Introduction

As organisms grow and develop, their cells undergo repeated rounds of division, each requiring the duplication of genomic DNA. During this process, replication errors, such as the misincorporation of the wrong DNA base or polymerase slippage (incorporating too many or too few bases), can introduce spontaneous mutations and indel frame shifts across the genome. Accumulation of mutations arising in this fashion collectively diminishes protein function and places a greater strain on cellular quality control systems; eventually, these deficiencies will compromise the these homeostatic processes [1,2,3,4]. Failure of these quality control mechanisms, especially those involved in DNA replication and repair, results in neoplasias that may develop into cancer [5,6,7]. Aberrations that compromise checkpoint mechanisms can accelerate the rate of mutation, often referred to as the mutator phenotype [8]. On this basis, we hypothesized that the increased rate of mutation in cancerous tissues may represent a relatively untapped resource for identifying naturally-occurring genetic variations that compromise cellular maintenance mechanisms.

Commonly known as the “powerhouse of the cell”, mitochondria are much more than just a site for energy production; they participate in a myriad of functions, including homeostatic regulation of calcium and iron [9], coordination of the production of reactive oxygen species (ROS) [10,11,12], provision of essential intermediates for and regulation of cholesterol biosynthesis, and fatty acid metabolism [13]. Mitochondria are critical for cellular health, and play key roles in the activation of apoptosis, whole cell autophagy, and other cell death mechanisms [14,15,16]. Critical pathways for maintaining mitochondrial homeostasis, including surveillance, repair, and recycling processes, have been identified within the past few decades; these gene networks often transcend this function to support cellular homeostasis more broadly, as evidenced by the fact that dysregulation of mitochondrial surveillance leads to proteostatic defects and disease [17,18,19,20,21,22]. Despite extensive work, gaps remain in our knowledge of how random mutations in genes responsible for healthy cellular maintenance can affect mitochondrial function.

The model host *C. elegans* has emerged as a critical tool for the discovery and understanding of gene networks for several reasons. First, the biology of this host makes it particularly effective for reverse genetics using RNAi feeding [23], and the development of well-established, high-throughput protocols for RNAi [24,25,26] have considerably expanded this utility. The transparency and invariant cell lineage of *C. elegans* have facilitated intricate study of cell division pathways, and the wide availability of fluorescently-tagged proteins that can be observed in intact, living samples has provided critical contributions to the study of cell biology.

In this study, we used the NCI-60 cancer cell database to identify genes frequently mutated in human cancers. Orthologous genes in *C. elegans* were identified and disrupted by RNA interference (RNAi) [27], and sensitivity to mitochondrial damage was subsequently evaluated. We obtained a set of 139 genes, spanning a wide variety of biological functions and pathways, that are involved in healthy mitochondrial function. To validate this conclusion, a subset of these genes was selected for further analysis. Disruption of each of these genes resulted in aberrant mitochondrial morphology and/or function, including activation of mitochondrial surveillance pathways. Disruption was also associated with pathological phenotypes, including muscle dysfunction, reduced fecundity, and increased protein aggregation. Finally, bioinformatic analysis determined that genes from this set were overrepresented in human disorders, supporting the functional significance of this set of genes. Our results indicate that these genes may merit further investigation.

## 2. Materials and Methods

### 2.1. Media and Strains

Standard conditions were used in the maintenance of *C. elegans* [28]. Worms were propagated on *Escherichia coli* OP50-seeded NGM plates at 20 °C, synchronized by hypochlorite bleaching, and hatched overnight at room temperature. Synchronized worms were maintained in S Basal at 15 °C prior to experimental use. The following *C. elegans* strains were used: N2 Bristol (wild-type), SS104 [*glp-4(bn2)*] [29], NVK90 [*pink-1*(*tm1779*); *houIs001* {*byEx655* (*Ppink-1*::PINK-1::GFP + *Pmyo-2*::mCherry)}] [30], SJ4103 [*zcIs14(Pmyo-3*::GFP^mt^)] [31], WY703 (*fdIs2* {*3XESRE*::GFP; *pFF4*[*rol-6*(*su1006*)]}) [32], SJ4100 {*zcIs13* [*Phsp-6*::GFP]} [30], SLR115 {*dvIs67* [*Ptbb-6*::GFP + *Pmyo-3*::dsRed]} [30], PE255 [*Psur-5*::luciferase::GFP + *rol-6*(*su1006*)] [33], ALF86 (*Pmyo-3*::Peredox::*unc-119*) [34], NL5901 [*pkIs2386* (*Punc-54*::α-synuclein::YFP + *unc-119*(+))] [35], JV1 {*jrIs1* [*rpl-17p*::HyPer+*unc-119*(+)]} [36], CL2166 {*dvIs19* [(*pAF15*)*gst-4p*::GFP::NLS]} [37], SJ4005 { *zcIs4* [*hsp-4*::GFP]} [38], GR2183 {*mgIs72* [*rpt-3p*::GFP + *dpy-5*(+)]} [39], and CF512 [*fer-15(b26);fem-1(hc17)*] [40]. For all RNAi experiments, worms were reared on NGM/Carbenicillin (Carb) (25 μg/mL)/IPTG(1 mM) plates containing *E. coli* HT115(DE3)-based RNAi strains for 60 h at 20 °C, unless otherwise noted. RNAi clones all came from the Ahringer or Vidal libraries and were sequenced for verification.

### 2.2. Precocious Mitophagic Activation (Primary Screening Assay)

Approximately 1000 L1 stage *Ppink-1*::PINK-1::GFP worms were dropped onto 6 cm NGM/Carb/IPTG, RNAi-seeded plates and incubated at 25 °C for 48 h. Each 6 cm plate was washed with S Basal, and 250 worms were transferred into two wells of a 96-well plate (500 worms total). The worms from the 96-well plates were sorted into a 384-well plate using a COPAS FlowPilot (FP) BioSorter and Large Particle (LP) Sampler (Union Biometrica, Holliston, MA, USA), with 20 worms per well. *E. coli* HT115 bacteria (food source, final OD600 = 0.1) and 7 mM sodium selenite were added to the 384-well plates. Brightfield and GFP images were taken every 3 h for 30 h using the Cytation5 Cell Imaging Multi-Mode Reader (BioTek Instruments, Winooski, VT, USA). GFP quantification was performed by using an image analysis pipeline previously established in CellProfiler software (Version v.10415) [24]. Gene knockdowns were counted as hits if the GFP level was 1.4 times that of vector control. At least three biological replicates were performed.

### 2.3. Sensitivity to Acute and Chronic Mitochondrial Damage (Secondary Screening Assays)

For measurement of sensitivity to acute mitochondrial damage, ~1000 L1 stage *glp-4(bn2)* worms were dropped onto 6 cm NGM/Carb/IPTG, RNAi-seeded plates and incubated overnight at 20 °C, then shifted to 25 °C for 48 h. Worms were placed into 384-well plates as described above. Next, 0.357 μM Sytox Orange stain (Invitrogen, Waltham, MA, USA) was added to each well and incubated for 5 h. Then 50 μM of the mitochondrial uncoupler carbonyl cyanide *m*-chlorophenyl hydrazone (CCCP) (Sigma, St. Louis, MO, USA) was added to each well. Brightfield and RFP-channel images were taken every 30 min for 12 h using the Cytation5 Cell Imaging Multi-Mode Reader (BioTek Instruments, Winooski, VT, USA). Fluorescence quantifications and the counting of fraction of dead worms were performed by using a CellProfiler pipeline. Gene knockdowns were counted as hits if the number of dead worms was 1.5 times that of the empty vector control. At least three biological replicates were performed.

For measurement of sensitivity to chronic mitochondrial damage, L1 stage *glp-4(bn2)* worms were dropped onto 6 cm NGM/Carb/IPTG, RNAi-seeded plates and incubated at 25 °C for 72 h. Worms were picked onto 35 mm OP50-seeded NGM plates supplemented with 100 μM of the iron chelator 1,10-phenanthroline (Sigma, St. Louis, MO, USA). Four days later, worms were scored and counted as dead if they did not respond to touch. Three biological replicates were performed; each biological replicate consisted of three technical replicates, each of 60 worms. RNAi knockdown of genes that showed a significant increase in death compared to the empty vector control were considered hits. Student’s *t*-test was used to calculate statistical significance.

### 2.4. Longevity Assay (Tertiary Screening Assay)

During the first iteration of screening (seed set determination), for measurement of longevity, *glp-4(bn2)* worms were grown to a young adult stage on RNAi or vector control plates as described above. Then they were placed on *E. coli* OP50 NGM plates and were scored every other day. Three biological replicates comprising three technical replicates each were performed (~150 worms/biological replicate). The log-rank test was used to determine statistical significance. For the second iteration of screening (set expansion), worms were grown and longevity plates were set as above, but survival on a single day, day 8, was scored. RNAi clones with survival of at least 90% of vector control were considered healthy (e.g., if 85% of *C. elegans* on vector RNAi were alive, RNAi with survival > 76.5% will be considered healthy).

### 2.5. Fluorescence Microscopy

To visualize morphology of the mitochondrial network, L1 stage *Pmyo-3*::GFP^mt^ worms were grown on 6 cm NGM/Carb/IPTG, RNAi-seeded plates and incubated at 20 °C for 60 h. The worms were then washed in S Basal, immobilized with 1.5% levamisole (Sigma, St. Louis, MO, USA), and pipetted onto agar pads. The largest center muscle was used for imaging and quantification in each worm to decrease variation due to worm position and mitochondrial content. At least 30 worms were used per condition per biological replicate, and three replicates were completed per condition. Images were acquired using a Zeiss Axio Imager M2 upright microscope with a Zeiss AxioCam 506 Mono camera and Zeiss Zen2Pro software (Carl Zeiss AG, Jena, Thuringia, Germany). Statistical significance was determined by the χ^2^- test.

To visualize α-synuclein aggregation, L1 stage *Punc-54*::α-synuclein::YFP worms were grown on 6 cm NGM/Carb/IPTG, RNAi-seeded plates and incubated at 20 °C for 72 h. The worms were then picked onto 6 cm *E. coli* OP50-seeded NGM plates and incubated at 20 °C for 48 h. Worms were then washed in S Basal, immobilized with 1.5% levamisole (Sigma, St. Louis, MO, USA), and pipetted onto agar pads. At least 25 worms were used per condition per biological replicate, and six replicates were completed per condition. Worms were visualized using a Zeiss Axio Imager M2 upright microscope with a Zeiss AxioCam 506 Mono camera and Zeiss Zen2Pro software (Carl Zeiss AG, Jena, Thuringia, Germany), and aggregates were manually counted.

### 2.6. Monitoring Mitochondrial Surveillance Pathway Activation 

To visualize the effect on mitochondrial surveillance pathways, L1 stage *3XESRE*::GFP (ESRE), *Phsp-6*::GFP (UPR^mt^), and *Ptbb*-6::GFP (MAPK^mt^) worms were grown on 6 cm NGM/Carb/IPTG, RNAi-seeded plates and incubated at 20 °C for 46 h. Each 6 cm plate was washed with S Basal and 100 worms were transferred into two wells of a 96-well plate (200 worms total per biological replicate). To induce ESRE activation, 25 μM rotenone was added. To induce UPR^mt^ and MAPK^mt^ activation, respective reporters were reared on *spg-7(RNAi)* and the experimental RNAi using a 1:3 ratio of *spg-7(RNAi)* (100 μL) to experimental RNAi (300 μL). *spg-7(RNAi)* is a very potent activator of these pathways, thus ¼ of the dose was sufficient for upregulation of the reporters. Brightfield and GFP images were taken every 1.5 h for 12 h using the Cytation5 Cell Imaging Multi-Mode Reader (BioTek Instruments, Winooski, VT, USA). GFP quantification was performed using the Gen5 software (Version 3.10).

### 2.7. Monitoring Activation of Non-Mitochondrial Surveillance Pathways

To visualize the effect on nonmitochondrial surveillance pathways, L1 stage worms carrying *gst-4*::GFP (oxidative stress), *hsp-4*::GFP (UPR^ER^), or *rpt-3*::GFP (proteasome) reporters [41,42,43] were grown on 6 cm NGM/Carb/IPTG, RNAi-seeded plates and incubated at 20 °C for 46 h. Each 6 cm plate was washed with S Basal and 100 worms were transferred into two wells of a 96-well plate (200 worms total per biological replicate). To induce stress in our *gst-4*::GFP, *hsp-4*::GFP, and *rpt-3*::GFP positive controls (empty vector RNAi), 60 μM juglone, 60 μM tunicamycin, or 20 μM bortezomib were added, respectively. Brightfield and GFP images were taken every 1.5 h for 12 h using the Cytation5 Cell Imaging Multi-Mode Reader (BioTek Instruments, Winooski, VT, USA). GFP quantification was performed using the Gen5 software (Version 3.10).

### 2.8. NADH/NAD^+^ Ratio Measurements

To measure the NADH/NAD^+^ ratio, L1 stage *Pmyo-3*::Peredox::*unc-119* worms were grown on 6 cm NGM/Carb/IPTG, RNAi-seeded plates and incubated at 20 °C for 60 h. The worms were then washed in S Basal and transferred to a 96-well plate (50 worms per well, 4 wells per RNAi condition). The plate was then loaded into the Cytation5 Cell Imaging Multi-Mode Reader (BioTek Instruments, Winooski, VT, USA), and fluorescence in GFP and RFP channels was obtained. The ratio was calculated based on the GFP and RFP fluorescence ratios.

### 2.9. ROS Measurements

To measure ROS, L1 stage *glp-4(bn2)* worms were grown on 6 cm NGM/Carb/IPTG, RNAi-seeded plates and incubated at 20 °C for 24 h, then shifted to 25 °C for 48 h. The worms were then washed in S Basal, and transferred to a 96-well plate (100 worms per well, 2 wells per RNAi condition) with 50 μM of DCFDA (ThermoFisher, Waltham, MA, USA). The plates were incubated in the dark at room temperature for 1 h, and then washed with S Basal to remove residual dye. The plate was then loaded into the Cytation5 Cell Imaging Multi-Mode Reader (BioTek Instruments, Winooski, VT, USA), and 485 nm excitation and 530 nm emission settings were used for fluorescence quantification.

### 2.10. Hydrogen Peroxide Measurements

To measure hydrogen peroxide, L1 stage *Prpl-17*::HyPer + *unc-119*(+) worms were grown on 6 cm NGM/Carb/IPTG, RNAi-seeded plates and incubated at 20 °C for 72 h. The worms were then washed in S Basal and transferred to a 96-well plate (100 worms per well, 2 wells per RNAi condition). The plate was then loaded into the Cytation5 Cell Imaging Multi-Mode Reader (BioTek Instruments, Winooski, VT, USA), and fluorescence was obtained with 405 nm and 495 nm excitation and 535 nm emission filters. Relative peroxide levels were calculated based on the ratio of emission fluorescence after individual stimulation at each of the two excitation parameters.

### 2.11. Flow Vermimetry

L1 stage N2 worms were grown on 6 cm NGM/Carb/IPTG, RNAi-seeded plates and incubated at 20 °C for 60 h. Worms were then washed with S Basal, transferred to 96-well plates, and stained with 5 μM of MitoTracker Green FM (Invitrogen, Waltham, MA, USA) for mitochondrial mass, or 4.5 μM MitoTracker Red CMXRos (Invitrogen, Waltham, MA, USA) for membrane potential in S Basal, and compared to no-stain controls. Fluorescence was measured with a COPAS FP BioSorter (Union Biometrica, Holliston, MA, USA), as previously described [44]. Three biological replicates were performed, and each consisted of two technical replicates, with 200 worms each. Worm size data were recorded by the COPAS FP BioSorter (Union Biometrica, Holliston, MA, USA) through time of flight and normalized to the empty vector control.

### 2.12. Oxygen Consumption

A total of 10,000 N2 worms were grown on 10 cm NGM/Carb/IPTG, RNAi-seeded plates and incubated at 20 °C for 68 h. Worms were washed in S Basal, and then counted and sorted using the COPAS FP BioSorter (Union Biometrica, Holliston, MA, USA). A maximum of 10,000 worms were used, with an average of 8000 worms per replicate, and the exact worm number was recorded. For digestion of bacteria, 30 min were allowed before measurement. Oxygen consumption was measured at 20 °C with a YSI Model 5300 Biological Oxygen Monitor and a YSI 5301 Clark-type oxygen electrode (Yellow Springs Instruments, Yellow Springs, OH, USA), and readings were continuously recorded for about 5–15 min, depending on rate. Oxygen consumption rate was measured from the slope of the straight section of the recorded graph and normalized to worm number; 5–7 replicates were completed for each condition.

### 2.13. Fecundity

To measure brood size, L1 stage *glp-4(bn2)* worms were grown on 6 cm NGM/Carb/IPTG, RNAi-seeded plates and incubated at 15 °C for 80 h. After that, individual young adult worms were picked onto 6 cm OP50-seeded plates (10 technical replicates) and shifted to 25 °C for the remainder of the brood size calculation. We acknowledge that this strain is temperature sterile, but this effect is only significant if worms are placed at the temperature-restrictive condition prior to reaching the young adult stage, so we expected to see no significant effect on the number of progeny. We used this strain to facilitate counting all the progeny on one plate without having to move the parental worm to a new plate each day. To address this limitation, we decided to verify our results from 2 different RNAi constructs, one that exhibited a significant decrease in brood size and another one that did not, compared to empty vector control, in a standard N2 strain and a *fer-15;fem-1* temperature-sterile mutant. For the *fer-15;fem-1* mutant, the assay was conducted in the same manner as with the *glp-4(bn2)* worms. The N2 strain was grown on 6 cm NGM/Carb/IPTG, RNAi-seeded plates and incubated at 20 °C for 60 h. Then individual young adult worms were picked onto 6 cm OP50-seeded plates (10 technical replicates) and kept at 20 °C for the remainder of the brood size calculation. Once L1 stage worms were seen on the plate with the parental worm, the parent was picked onto a fresh OP50-seeded plate, and this continued until all the progeny were scored (normally across a 5-day period).

### 2.14. Statistical Analysis

Unless stated otherwise, all experiments were performed in three biological replicates. One-way ANOVA analysis was used to calculate significance, unless otherwise stated. Dunnett’s post hoc analysis was used to calculate *p*-values between each condition and the control group for ANOVA testing. On the graphs, statistical significance is represented as follows: * *p* < 0.05, ** *p* < 0.01, and *** *p* < 0.001.

## 3. Results

### 3.1. Identification of a Gene Set Associated with Increased Sensitivity to Mitochondrial Damage

**Figure 1:**
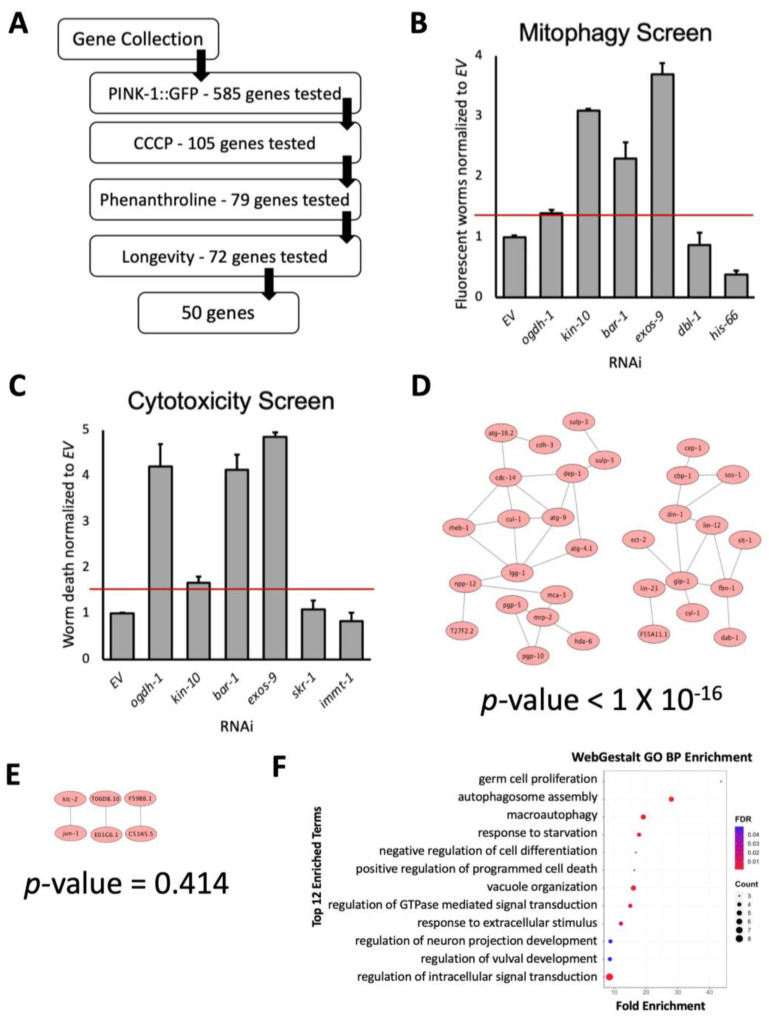
Identification of a gene set underlying mitochondrial health. (**A**) Flowchart of testing process for genes identified from the NCI-60 hit list. The flowchart indicates the number of genes tested at each stage. (**B**) A chart illustrating the 1.4-fold cutoff (indicated by a red line) used to obtain the hits from screening for mitophagic activation. (**C**) A chart showing the 1.5-fold cutoff (indicated by a red line) used to identify hits in the cytotoxicity screen. (**D**) Visualization of the initial 50 gene ‘seed set’, showing 38 connections between the genes. (**E**) Connectivity of one representative set (of 100 sets) of 50 randomly-selected genes from the *C. elegans* genome, showing a total of three connections. *p*-values (**D**,**E**) were calculated by the WormNetV3 database and include a Bonferroni correction. (**F**) Bioinformatic analysis of the 50 gene set showed enrichment for germ cell proliferation, negative regulation of differentiation, and autophagic flux (autophagosome assembly, macroautophagy, response to starvation, etc.).

Using the rationale that cancer mutations are sporadic (see Introduction), we used the NCI-60 cancer database, comprising 60 different tumor cell lines spanning a variety of tumor types, to look for genes frequently mutated in multiple cancer cell lines. The Catalogue of Somatic Mutations in Cancer (COSMIC) and the Cancer Cell Line Encyclopedia (CCLE) databases were used to identify 15,856 genes with mutations in at least one of the 60 cell lines. To improve robustness, any gene that was not mutated in at least 10 cell lines originating from at least three different tissues was removed, leaving us with 385 frequently-mutated genes. The list was also supplemented with genes that were mutated in at least three of the eight NCI-60 cell lines that were most sensitive to mitochondrial damage (LE:SR, LE:CCRF-CEM, ME:LOX IMVI, LE:MOLT-4, CNS:U251, BR:MCF7, LE:HL-60(TB), LC:NCI-H460) [45,46]. This added a total of 251 genes to the list and was expected to enrich the list for genes that played a role in sensitivity to mitochondrial damage. Finally, a small subset of 20 genes, primarily involved in signal transduction, were manually added, yielding a final list of 656 genes. Since we hypothesized that mutations accumulating during cellular senescence would generally reduce protein function, RNAi was chosen as a rapid method for carrying out reverse genetics analysis on these genes. Bioinformatic tools, such as Ortholist and Ensembl [47,48], were used to identify putative *C. elegans* orthologs for each human gene, yielding 585 *C. elegans* orthologs for testing. A testing pipeline was developed that would allow identification of genes that play a role in mitochondrial health (Figure 1A).

Mitochondrial autophagy (mitophagy) is a critical response to mitochondrial damage, and is crucial for maintaining mitochondrial health [49]. To screen for genes involved in this process, we utilized a reporter for the PINK1/Parkin pathway, which is a main regulator of mitophagic activation [50]. Under normal conditions, PINK-1 (the *C. elegans* homolog of the mammalian PINK1 protein) *is* efficiently imported into mitochondria, where it is rapidly degraded by matrix-resident proteases. Mitochondrial damage limits PINK-1 internalization, stabilizing the protein on the mitochondrial surface. This allows it to auto-crossphosphorylate, recruiting Parkin to help initiate mitophagy. RNAi was used to knock down each of the genes in a *C. elegans* reporter strain carrying a *Ppink*-1::PINK-1::GFP translational fusion. Because of PINK-1′s normal turnover, GFP should only be observed if it is excluded from the mitochondria, stabilizing the protein. On this basis, increased GFP fluorescence was used as a proxy for mitophagic initiation. Synchronized L1 worms were dropped onto *E. coli* expressing dsRNA of the gene of interest and incubated for 48 h at 25 °C. Young adult stage worms were then exposed to sodium selenite (a known inducer of mitophagy [51,52]). Brightfield and fluorescence images were collected every 2 h from 10 to 24 h after sodium selenite exposure to observe PINK-1::GFP accumulation. Images were then analyzed by an unbiased, automated pipeline to compare fluorescence levels from empty vector (*EV)* controls to experimental RNAi mutants. Specifically, PINK-1::GFP accumulation was compared once 10–20% of vector control worms exhibited clear signs of PINK-1::GFP accumulation. This provided some range for increased PINK-1::GFP accumulation, indicating that the gene disruption caused precocious mitophagic activation. Knockdowns that exhibited at least 1.4-fold increase (to account for the variability of 20% from average) in PINK-1::GFP fluorescence were considered hits in the primary assay (Figure 1B, Table S1). This cutoff also generally passed the threshold for significance using other statistical analyses, such as ANOVA and Student’s *t*-test. As variability slightly differed from plate to plate, the minimum statistically significant fold-change varied between plates. Using a set fold-change cutoff provided us with a standardized measure for primary hit selection.

A total of 105 genes (16% of the original set) passed this primary cutoff and were moved to a secondary screen. We hypothesized that compromising removal of damaged mitochondria would increase sensitivity to mitochondria-affecting compounds. RNAi was used to test whether knockdown increased sensitivity to acute or chronic mitochondrial stress. For these assays, synchronized L1, temperature-sensitive, *glp-4(bn2ts)* worms were reared on RNAi for each of the 105 genes. The *glp-4(bn2)* allele causes a temperature-dependent sterility that simplifies lifespan and survival assays by removing changes associated with reproduction.

For acute mitochondrial damage, young adult worms were sorted into 384-well plates and exposed to the mitochondrial uncoupler carbonyl cyanide *m*-chlorophenyl hydrazone (CCCP), which depolarizes the mitochondrial membrane and causes death [53]. Starting 6 h after exposure to CCCP, survival was assayed every 30 min for 6 h using the vital dye Sytox Orange. Genes whose disruption increased death by at least 1.5-fold compared to vector control were considered hits (Figure 1C, Table S2). Since this assay had more variability than the first assay, a higher cutoff of 1.5-fold was chosen to minimize the number of false-positives (type I error). As with the primary screen, this cutoff generally agreed with the outcomes of ANOVA and Student’s *t*-test.

A total of 79 genes (75% of the genes tested) passed this screen and were tested in an orthogonal screen for increased sensitivity to chronic mitochondrial damage. Young adult *glp-4(bn2)* worms were placed on NGM plates containing 100 µM 1,10-phenanthroline. Phenanthroline is a small-molecule iron chelator that damages mitochondria and kills *C. elegans* [54,55,56]. Survival was scored daily until vector controls reached at least 90% death. Log-rank tests were used to identify genes whose disruption significantly reduced host survival. A total of 72 of the 79 genes (~90%) passed this test. As analysis showed a very strong correlation between acute and chronic mitochondrial toxicity, the chronic exposure assay was dropped for subsequent iterations of screening.

To eliminate the possibility that the increased sensitivity observed was due to the target genes being required for survival under normal conditions, lifespan assays were performed with RNAi of the 79 genes in *glp-4(bn2)* worms. Disruption of 29 genes (37%) reduced 10-day survival below 90% of vector control, and hence were removed from the list for potential nonspecificity. After these tests were completed, an initial seed list of 50 genes remained (see Table S3 for the gene list).

Gene interaction assessment tools, such as String and WormNet (a network-assisted predictive tool that mines dozens of connection types, including in vivo and in vitro protein–protein interactions, genetic interactions, interactions between orthologs, cocitations, etc. [57,58]), identified 38 connections amongst these 50 genes (Figure 1D). This connectivity was significantly greater (*p*-value < 1.00 × 10^−16^) than was observed on average for 100 equivalently-sized sets of randomly chosen genes (*p*-value = 0.414) (Figure 1E). (Connectivity here refers to actual connections observed based on the data mining parameters used by String and WormNet, which include experimentally determined interactions, data from curated databases in other species, and also predicted from coexpression, co-occurrence, gene neighborhoods, etc.; it does not refer to shared phenotypes or proximity to each other in biological function.) Predicted and annotated gene functions for this set of 50 genes included membrane transport, germ cell proliferation, negative regulation of differentiation, and autophagic flux (autophagosome assembly, macroautophagy, response to starvation, etc.), indicating that our gene list spanned a variety of biological functions (Figure 1F).

### 3.2. Expansion of the Gene Set Identifies Genes That Impact Mitochondrial Health

Disruption of every gene in the seed set increased the sensitivity of *C. elegans* to mitochondrial damage. However, the set was too small to identify meaningful trends. To expand this list, WormNet [58] was used to identify additional genes that were significantly (*p*-value < 0.05) associated with the seed set, yielding ~400 additional candidate genes (Table S4). WormNet output assigned each gene an individual score based on the number of connections it had with the genes in the seed set, and the strength of these connections, based on evidence from data mining. The higher the score, the more significantly the gene was predicted to be connected to the network. We used a score cutoff of six for network expansion. As these additional genes were predicted to be related to the seed set, we anticipated that disrupting many of them would also increase sensitivity to mitochondrial damage. Approximately 20 genes whose mutations were reported as lethal in WormBase were removed from the test list, as their disruption is likely to cause pleiotropic effects, such as cell division arrests or dramatic metabolic dysfunctions. As these effects could compromise aspects of cellular activity that extended far beyond mitochondrial function, the effect of disrupting these genes would be nonspecific, and would not meaningfully indicate mitochondrial disruption. RNAi was used to test each of the remaining genes for PINK-1::GFP accumulation, increased sensitivity to acute mitochondrial poison, and lifespan.

**Figure 2:**
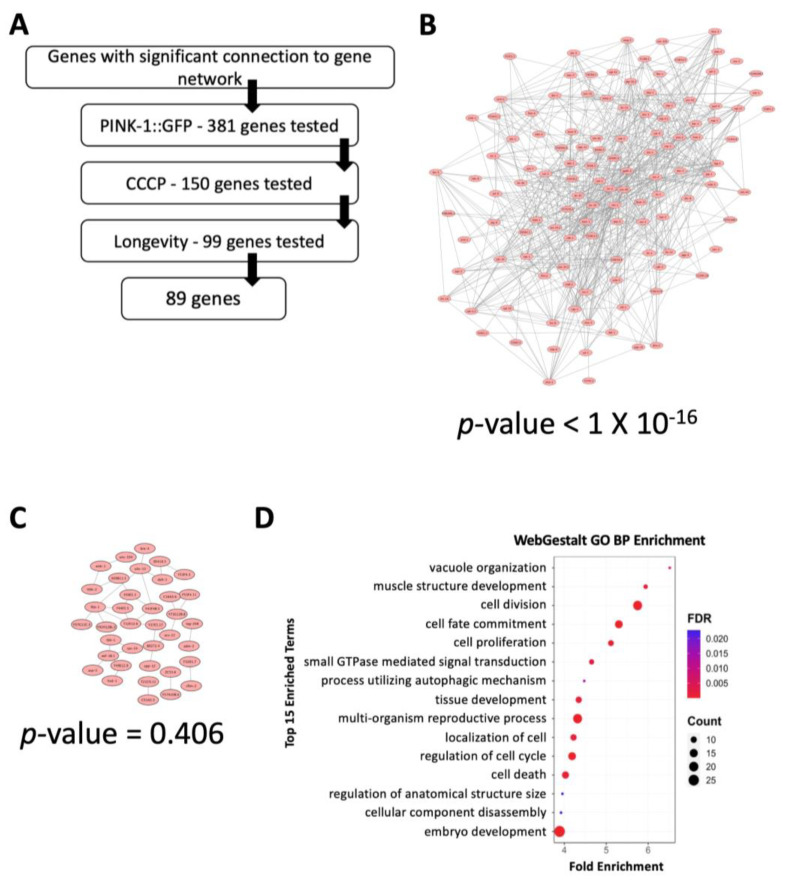
Refinement of the mitochondrial health gene set. (**A**) Flowchart for testing the 381 new genes identified by their connection to the seed set. (**B**) Connectivity of the expanded 139 gene set. (**C**) Connectivity of one representative set (of 100 sets) of 140 randomly selected genes from the *C. elegans* genome, showing a total of ~40 connections. *p*-values (**B**,**C**) were calculated by the WormNetV3 database and include a Bonferroni correction. (**D**) Bioinformatic analysis of the 139 gene set showed enrichment for cell proliferation and development, as well as signal transduction.

Disruption of 89 of the 381 expansion genes increased mitochondrial sensitivity without overt effects on lifespan in the absence of mitochondrial stress (Figure 2A). These genes, when knocked down, increased sensitivity to mitochondrial damage and a decrease in lifespan upon mitochondrial disruption. In contrast, no change in lifespan was seen under normal conditions. These genes were added to the initial 50, yielding an expanded set of 139 genes. As expected, the number of connections between these genes was dramatically higher than for identically-sized, randomly-selected sets of genes (compare Figure 2 panels 2B and 2C; an average random gene set from ~100 tested is shown in Figure 2C). Using WebGestalt [59], we discovered a six-fold enrichment in genes associated with cell division (Figure 2D). Although this enrichment is likely partly explained by our use of cancer cells for the initial identification, our results also suggested a connection between cell cycle genes and mitochondrial dysfunction. Genes associated with the ErbB (e.g., *mkk-4*, *plc-3*, *nck-1*, *sem-5*, *ced-2*, and *sos-1*) and Wnt (e.g., *kin-3*, *kin-10*, *cul-1*, *lin-23, pop-1*, *cbp-1*, *bar-1*, *cyd-1*, and *rho-1*) signaling pathways were also enriched. Both of these pathways are connected to cancer [60,61]. Interestingly, little is known about how these pathways contribute to mitochondrial health, despite extensive study.

### 3.3. Disruption of a Validation Subset Impairs Mitochondrial Function

A *k*-means clustering program was used to group the 139-gene set into eight clusters, each cluster comprising genes with the most significant connections to each other as compared to the other genes in the network (Figure S1). To examine the consequences of gene knockdown on mitochondrial function, the output from *k*-means clustering was used to select a panel of eight genes with diverse functions and cellular locations (*ogdh-1*, *kin-10*, *ego-2*, *bar-1*, *pps-1*, *exos-9*, *cdc-37*, and *cul-1*), which were selected for further study (Table 1). As shown in the table, three out of the eight genes were known to have direct interactions with mitochondria; the others did not. Instead, these other genes play roles in a variety of biological functions, such developmental signaling, exonuclease activity, and protein degradation. These genes were chosen as representing the gene set to study the effects of gene disruption across a variety of gene functions.

**Figure 3:**
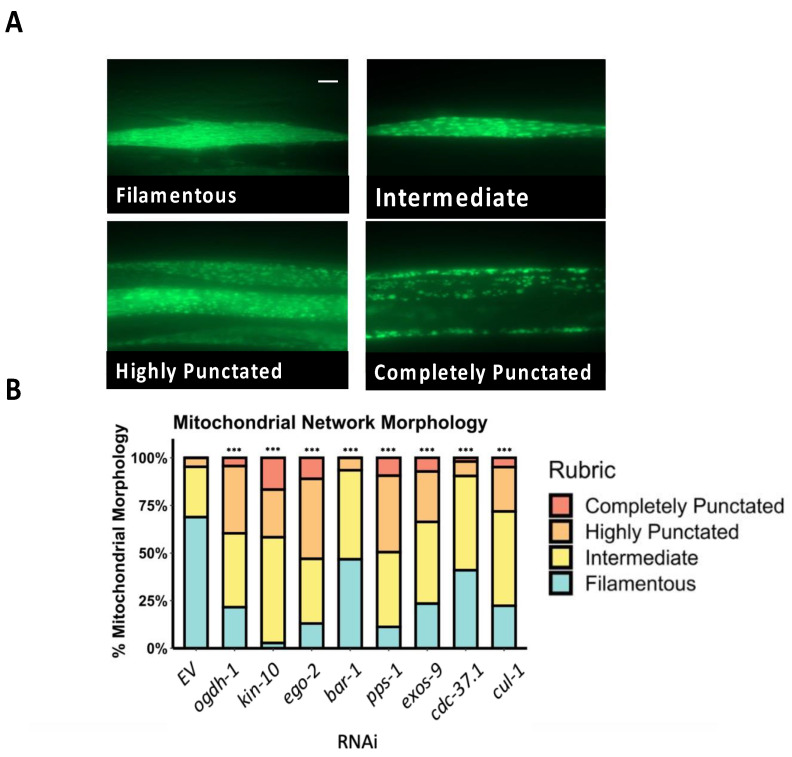
Genes in the mitochondrial health gene set support mitochondrial network connectivity. (**A**) Fluorescence micrographs of mitochondrially-targeted GFP (GFP^mt^) expressed in body wall muscle cells showing four different qualitative states. Shown are filamentous (top left), intermediate fragmentation (upper right), highly punctate (bottom left), and completely punctate (bottom right). Scale bar represents 5 μm. (**B**) Quantification of fragmentation state of worms expressing GFP^mt^ in body wall muscles after RNAi targeting selected genes. Approximately 90 worms were examined for each genotype. All genes showed an increase in mitochondrial fragmentation after RNAi. Statistical significance was determined by χ^2^ analysis, *** *p* < 0.001.

As a first step in our testing, we used a *C. elegans* reporter strain carrying mitochondrially-targeted GFP expressed in body wall muscle cells (referred to herein as GFP^mt^) to visualize mitochondria. Body wall muscle cells in *C. elegans* possess a characteristic network of mitochondria, appearing as long, branched tubules; mitochondrial damage causes this network to fragment (see Figure 3A). For this test, synchronized L1 GFP^mt^ worms were reared on RNAi targeting each of the eight genes. Once the worms reached the young adult stage, fluorescence microscopy was used to visualize and quantify mitochondrial fragmentation compared to *EV*. RNAi for each of the genes resulted in significant mitochondrial fragmentation (*p*-value < 0.001), supporting the conclusion that they are associated with mitochondrial maintenance (Figure 3B).

**Figure 4:**
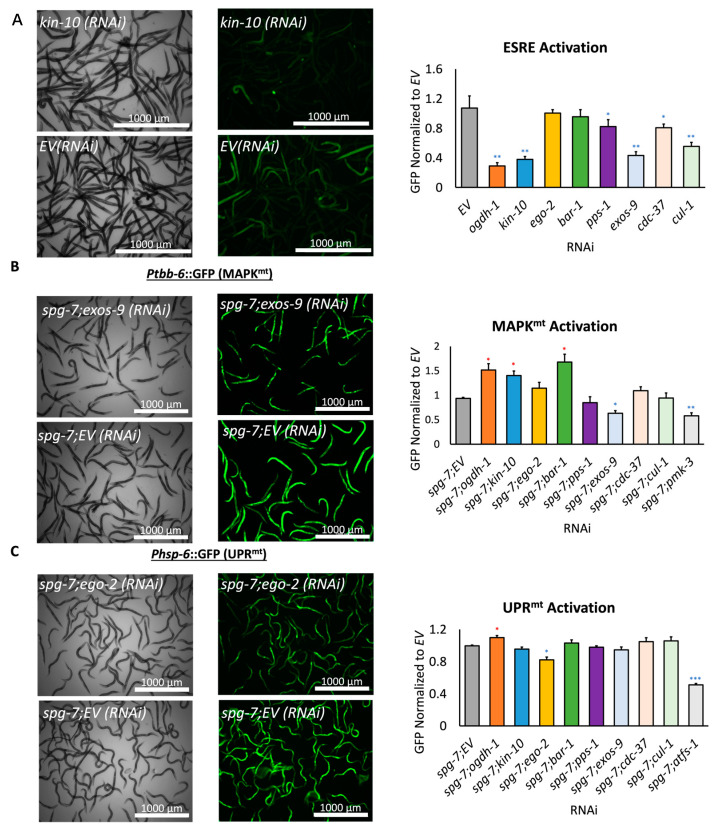
Genes in the mitochondrial health gene set support healthy mitochondrial surveillance. (**A**) Brightfield and GFP images of the *3XESRE*::GFP reporter are quantified to compare ESRE expression of the validation RNAi panel. (**B**) Brightfield and GFP images of the *Ptbb-6*::GFP reporter are quantified to compare MAPK^mt^ expression across the RNAi validation panel, with *pmk-3* being the positive control. (**C**) Brightfield and GFP images of the *Phsp-6*::GFP reporter are quantified to compare UPR^mt^ expression across the RNAi validation panel, with *atfs-1* being the positive control. Statistical significance in all panels was calculated using one-way ANOVA analysis, followed by a Dunnett’s post hoc test. Statistical significance in all panels was calculated using one-way ANOVA analysis, followed by a Dunnett’s post hoc test. * *p* < 0.05, ** *p* < 0.01, *** *p* < 0.001. * indicates significant increase compared to *EV*, * indicates significant decrease compared to *EV*.

While the mitochondrial fragmentation data were indicative of mitochondrial dysfunction, we wanted to evaluate the effect of knocking these genes down on mitochondrial maintenance by testing for activation of three mitochondrial surveillance pathways. Reporters were used to assess activation of the ESRE (*3XESRE*::GFP, three tandem repeats of the ESRE motif fused to GFP), UPR^mt^ (*Phsp-6*::GFP, GFP driven by the promoter of *hsp-6*, a marker of UPR^mt^), and MAPK^mt^ (*Ptbb-6*::GFP, GFP driven by the promoter of *tbb-6*, a marker of MAPK^mt^) pathways [62,63,64]. Synchronized L1 worms carrying each reporter were spotted onto RNAi of the eight genes in the panel, reared to the young adult stage, and then GFP fluorescence was quantified after induction of pathways with rotenone for ESRE or *spg-7(RNAi)* for UPR^mt^ or MAPK^mt^, along with control RNAi for UPR^mt^ (*atfs-1*) or MAPK^mt^ (*pmk-3*) [62,65,66]. It is worth noting that there is not a well-established regulator for ESRE, so a control was not available for this assay. Each RNAi showed disrupted regulation of at least one of the mitochondrial surveillance pathways (Figure 4).

**Figure 5:**
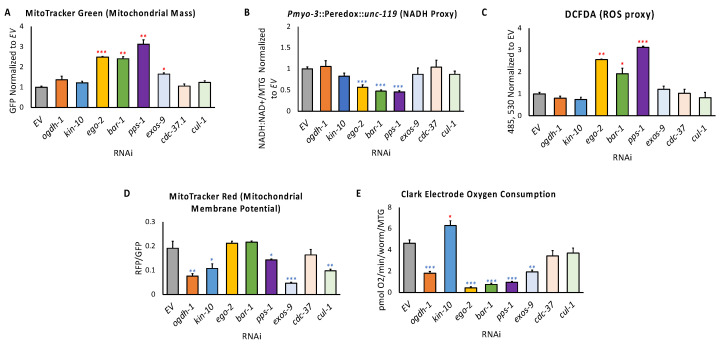
Genes in the mitochondrial health gene set support mitochondrial health. (**A**) Bar graph of mitochondrial mass, as measured by mitochondrial staining with MitoTracker Green FM in young adult worms reared on RNAi targeting the indicated gene. Fluorescence was measured using flow vermimetry. All subsequent mitochondrial functional assays were normalized to mitochondrial mass. (**B**) Bar graph of steady-state ratio of NADH–NAD+ using a conformation-dependent fluorometric reporter. Young adult worms carrying the reporter were reared on the indicated RNAi constructs. (**C**) Bar graph of steady-state reactive oxygen species, as measured by conversion of H_2_DCFDA to a fluorescent state in young adult worms reared on RNAi targeting the indicated gene. Fluorescence was measured using Cytation5 Cell Imaging Multi-Mode Reader. (**D**) Bar graph of mitochondrial membrane potential, as measured by the dye MitoTracker Red CMXRos in young adult worms reared on RNAi targeting the indicated gene. Fluorescence was measured using flow vermimetry. (**E**) Bar graph of basal oxygen consumption in young adult worms reared on RNAi targeting the indicated gene as measured by a Clark electrode. Statistical significance in all panels was calculated using one-way ANOVA analysis followed by a Dunnett’s post hoc test. * *p* < 0.05, ** *p* < 0.01, *** *p* < 0.001. * indicates significant increase compared to *EV*, * indicates significant decrease compared to *EV*.

Mitochondrial mass was measured using the fluorescent dye MitoTracker Green FM [44]. Mitochondrial mass was increased over two-fold in *ego-2(RNAi)*, *bar-1(RNAi)*, and *pps-1(RNAi),* and slightly in *exos-9(RNAi)* (Figure 5A). It is worth noting that *ego-2(RNAi)* and *pps-1(RNAi)* exhibited severely fragmented mitochondrial networks (see Figure 3B), often interpreted as an imbalance between mitochondrial fission and fusion. This imbalance is known to reduce mitochondrial efficiency [67], and increased mitochondrial mass may be an attempt by the worm to compensate for reduced mitochondrial function.

In all subsequent experiments testing mitochondrial function, outcomes were normalized to mitochondrial mass to obtain “per mitochondria” values. Graphs with values not normalized to mitochondrial mass for these measurements are available in Figure S2.

Redox reactions, such as the oxidation of NADH to NAD^+^, are a hallmark of healthy mitochondrial function. We used the Peredox reporter, which fluoresces due to an NADH-dependent conformational change, to obtain a semiquantitative readout of cellular reductive potential. Synchronized L1 worms carrying the reporter were spotted onto RNAi of the eight genes in the panel and reared to the young adult stage, and then NADH levels were determined. *ego-2(RNAi)*, *bar-1 (RNAi)*, and *pps-1(RNAi)* disrupted redox status, as demonstrated by the decrease in Peredox fluorescence (Figure 5B).

Normal function of the electron transport chain produces a certain amount of ROS. Cells must detoxify these reactive metabolites to limit widespread damage to biomacromolecules. Synchronized larvae were spotted on RNAi and allowed to develop to young adulthood. Steady-state ROS levels were measured by staining with the ROS-reactive dye DCFDA, and fluorescence (normalized to worm size) was measured using a Cytation5 Multi-Mode reader. DCFDA fluorescence is an efficient method for measuring ROS in *C. elegans* due to the permeability of the cuticle and minimal signal loss due to the transparency of the organism [68,69,70,71]. Three conditions, *ego-2(RNAi)*, *bar-1(RNAi)*, and *pps-1(RNAi)*, showed increased ROS (Figure 5C).

We also used the HyPer reporter [36] to assess hydrogen peroxide levels. We observed no significant differences in hydrogen peroxide levels across the RNAi panel when compared to the empty vector control (Figure S3). If we were to normalize peroxide levels to mitochondrial mass, *ego-2(RNAi)*, *bar-1(RNAi)*, and *pps-1(RNAi)* exhibit a significant decrease in hydrogen peroxide levels. However, this could be an artifact due to the very low signal from the HyPer reporter, so normalization was not performed.

Mitochondrial membrane potential was also measured, using the fluorescent dye MitoTracker Red [44]. Mitochondrial membrane potential was significantly decreased in five out of eight RNAi conditions (Figure 5D).

Mitochondria utilize oxygen as the terminal electron acceptor in the electron transport chain. To assess mitochondrial function, oxygen consumption was measured after gene disruption using a biological oxygen monitor and a Clark-type oxygen electrode. Oxygen consumption was measured continuously for 5–15 min, and the basal oxygen consumption rate was derived from the slope of the recorded graph and normalized to worm count and mitochondrial mass. RNAi targeting *ogdh-1*, *ego-2*, *bar-1*, *pps-1*, and *exos-9* significantly reduced oxygen consumption. In contrast, oxygen consumption was significantly increased for *kin-10(RNAi)* (Figure 5E). A summary of these phenotypes with fold-change compared to the empty vector control is provided in Table 2.

### 3.4. Disruption of Novel Mitochondrial Maintenance Genes Affects Health in C. elegans

**Figure 6:**
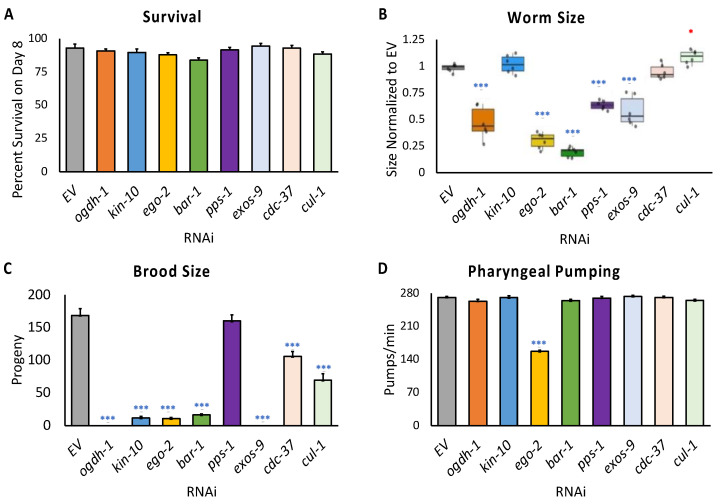
Genes in the mitochondrial health gene set support organism-level health parameters. (**A**) Bar graph of 8-day survival of worms reared on RNAi targeting the indicated gene. (**B**) Quantification of length of young adult worms reared on RNAi targeting the indicated gene. Worm length was measured using flow vermimetry. Circles represent average size per biological replicate. (**C**) Bar graph of worm fecundity in adult worms reared on RNAi targeting the indicated gene. (**D**) Bar graph of pharyngeal pumping rates for young adult worms reared on RNAi targeting the indicated gene. Statistical significance was calculated using one-way ANOVA analysis followed by Dunnett’s post hoc test. * *p* < 0.05, *** *p* < 0.001. * indicates significant increase compared to *EV*, * indicates significant decrease compared to *EV*.

To test specificity of these genes for mitochondrial surveillance, three surveillance systems for other cellular targets were analyzed: oxidative stress (*gst-4*), UPR^ER^ (*hsp-4*), and proteasomal stress (*rpt-3*). Knockdown of RNAi in the validation subset exhibited no activation in these surveillance pathways (Figure S4). In addition, preliminary testing of these genes indicated that they did not compromise the general health of the worms. A longer test showed that RNAi of these eight genes also did not adversely affect lifespan (Figure 6A). However, we wanted to assess the effects of gene disruptions across a broader array of organismal phenotypes. That could provide valuable information on the larger effect of mitochondrial dysfunction caused by decreased expression of these genes. Toward this end, we measured worm size, fecundity, and pharyngeal pumping.

*C. elegans* body size was determined by rearing synchronized L1 larvae on RNAi targeting each gene, and then using the COPAS FP BioSorter (Union Biometrica, Holliston, MA, USA) to measure adults using time-of-flight data. Five of the RNAi conditions reduced adult worm size, including *ogdh-1*, *ego-2*, *bar-1*, and *pps-1*, which were the most frequently affected in mitochondrial function assays (Figure 6B). Worms reared on *exos-9(RNAi)* also had a reduced adult size.

Mitochondrial damage negatively affects fecundity as well [72]. To determine whether these genes have a role in this process, synchronized L1 *glp-4(bn2)* worms were reared on RNAi targeting the eight genes in the panel and allowed to develop to young adulthood at permissive temperatures. Individual worms were then picked onto fresh OP50-seeded plates and shifted to a nonpermissive temperature to sterilize the offspring. Brood sizes were scored across a 5-day span, which is typically sufficient for worms to produce most of their offspring. Interestingly, only worms reared on *pps-1(RNAi)* retained normal fecundity (Figure 6C). This result ran somewhat counter to its otherwise generally strong impact on mitochondrial phenotypes. The results were also consistent with parallel experiments using N2 worms and *fer-15*;*fem-1* sterile mutants (Figure S5).

Finally, worms reared on each RNAi strain were observed under a dissecting microscope to score pharyngeal pumping (the number of pumps per minute was manually counted). Pumping was only significantly decreased in *ego-2(RNAi)*, suggesting that the mitochondrial dysfunction caused by gene knockdown did not compromise pharyngeal pumping, so food consumption was likely unchanged in these phenotypes (Figure 6D).

### 3.5. Genes from the Set Show an Increase in α-Synuclein Aggregation, a Feature of Parkinson’s Disease

**Figure 7:**
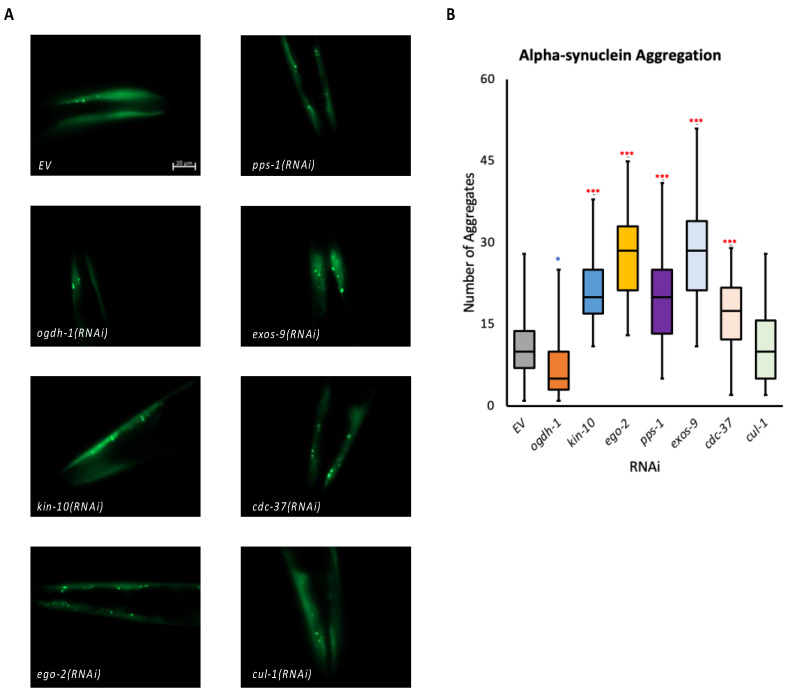
Genes in the mitochondrial health gene set reduce proteostatic aggregation. (**A**) Representative fluorescence micrographs of a transgenic *C. elegans* strain containing an α-synuclein::YFP protein in worms reared on RNAi targeting the indicated gene. (**B**) Quantification of α-synuclein::YFP aggregates in young adult worms reared on the indicated RNAi. Statistical significance was calculated using one-way ANOVA analysis followed by a Dunnett’s post hoc test. * *p* < 0.05, *** *p* < 0.001. * indicates significant increase compared to *EV*, * indicates significant decrease compared to *EV*.

Mitochondrial dysfunction has been implicated in neurodegenerative diseases, and genes related to Alzheimer’s and Parkinson’s disease are also found within our gene set. For this reason, we tested the effects of our novel mitochondrial maintenance genes on neurodegeneration. One staple of Parkinson’s disease (PD) is the aggregation of α-synuclein in the form of Lewy bodies found in the brains of many patients with PD [73]. We used a reporter strain that contains a fragment of the human α-synuclein protein fused to fluorescent reporter (YFP) expressed under control of the muscle-specific promoter P*unc-54* [35] to test the effects of RNAi knockdown on aggregate formation. Our results showed a significant increase in the number α-synuclein aggregates across most of the genes tested, except for *ogdh-1(RNAi)* and *cul-1(RNAi)* (Figure 7).

### 3.6. Pathways Related to Human Disease and Biological Processes Related to Cellular Division Are Overrepresented Amongst Human Orthologs

**Figure 8:**
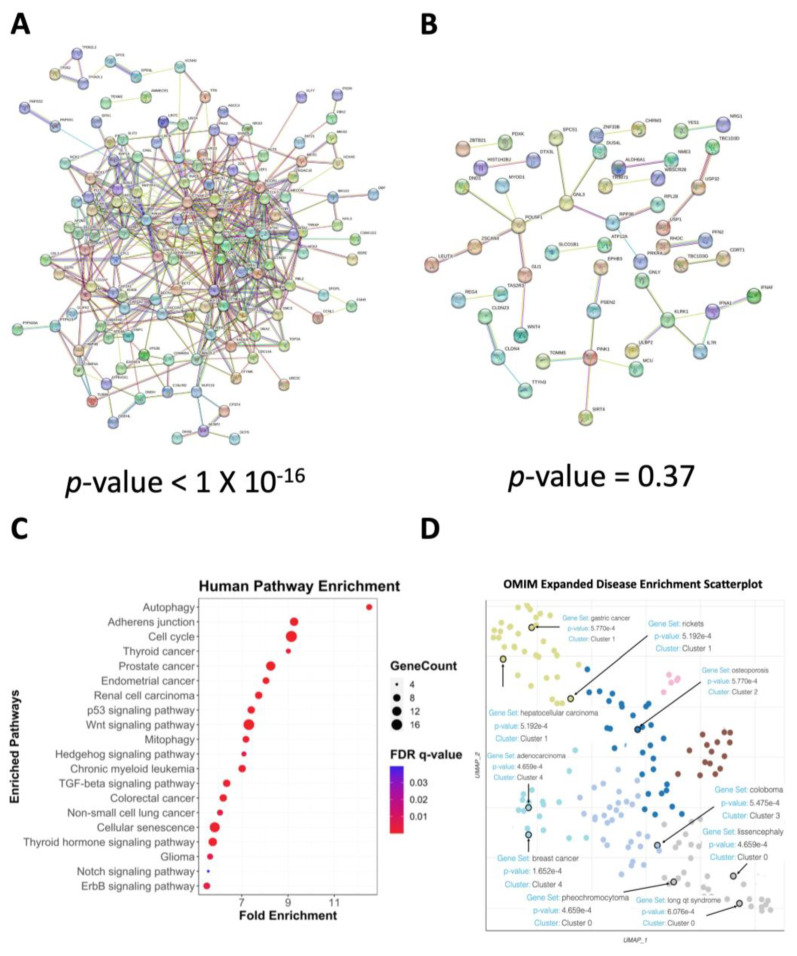
Human orthologs of the *C. elegans* mitochondrial health gene set illustrate increased co-association and are associated with disease. (**A**) A set of human genes comprising 153 orthologs of the *C. elegans* mitochondrial health gene set exhibits significant connectivity. (**B**) Connectivity of one representative set (of 100 sets) of 153 randomly selected genes from the human genome, showing a total of ~40 connections. *p*-values (**B**,**C**) were calculated by the STRING database. (**C**) Bioinformatic analysis of gene function for the human genes shows strong enrichment for autophagy and mitophagy, as well as cancer and cell division. Several pro-growth signaling pathways (such as p53, Wnt, Hedgehog, TGF-β, Notch, and ErbB signaling pathways) are also enriched. (**D**) Analysis of human orthologs also shows enrichment for a variety of diseases.

To extend the utility of our gene set, we looked for putative human orthologues of the 139 genes in the expanded set. Since it is common for gene duplication events to have expanded gene families in humans compared to *C. elegans*, a combination of tools, such as OrthoList, DIOPT, and Wormbase [47,74,75], and manual curation were used for assigning putative orthology. We attempted to strike a balance between thoroughness and not including an overabundance of genes with several highly similar co-orthologs (e.g., histone proteins, certain HSPs, etc.), so no more than two human genes with the greatest mutual similarity were considered putatively co-orthologous to each *C. elegans* gene (Table S5). The resulting human gene set comprised 156 genes with 503 connections (*p*-value < 1.00 × 10^−16^) (Figure 8A). A pool of 156 random genes from the human genome generated a gene set with only 37 connections (*p*-value = 0.37) on average across 100 iterations (Figure 8B). Gene ontology enrichment analysis using the KEGG database via WebGestalt identified several overrepresented categories, including cell cycle, signaling pathways (e.g., Wnt, Hedgehog, Notch), and autophagy (Figure 8C). Given that cancer cells were used for the identification of the genes tested for the initial seed set, the presence of these pathways was relatively unsurprising. However, this does not explain why so many of the identified genes disrupted mitochondrial function when expression was knocked down. It suggests that the identified genes may play unappreciated roles in mitochondrial function as well, providing a tantalizing avenue for research.

The presence of various diseases in the previous enrichment analysis led us to conduct an enrichment analysis focused solely on disease. For this bioinformatic analysis, we used the OMIM (Online Mendelian Inheritance in Man) expanded database which contains extensive data on human genes and their phenotypes, and can be filtered by subtype, including disease [76]. Using the Enrichr analysis tool along with the Enrichr Appyter function, we generated a scatterplot of diseases that were enriched in our gene set (Figure 8D). The diseases were clustered by genetic similarity, and we also obtained a list of the enriched diseases along with significance scores (Table S6). Even though the initial gene list was derived from mutations in cancer cell lines, the list of enriched diseases from our final gene set included a wide range of diseases, such as lissencephaly, osteoporosis, and coloboma. Further study of this gene set may potentially uncover novel mechanisms for mitochondrial homeostasis and its relevance in these diseases.

## 4. Discussion

In this manuscript we identified a set of 139 genes that are important for mitochondrial maintenance, as evidenced by gene knockdowns compromising mitochondrial function and increasing sensitivity to mitochondrial damage. This provides the first indication of this role for many of these genes and, in a broader context, validates the idea of studying frequently occurring mutations to identify underexplored genes related to mitochondrial maintenance.

Recent studies have discovered the role of mitochondria-related genes essential for tumor survival and genes that sense mitochondrial dysfunction using genome-wide CRISPR screens [77,78], validating the idea of interdependence of mitochondrial and cellular homeostasis. The first study discovered multitiered mechanisms for mTORC1 to surveil mitochondrial dysfunction [77]. The latter identified a relationship between OXPHOS genes and tumor cell growth in hypoxia and normoxia [78]. However, experiments performed under different conditions will likely identify different sets of genes of interest. For example, there is little overlap in targets between these two papers. This supports our assertion that the role of cytoplasmic genes on mitochondrial function merit study to identify new maintenance mechanisms.

Of the eight genes tested in the panel, *pps-1(RNAi)* showed the greatest increase in mitochondrial mass. This increase may be an indicator of accumulated mitochondria due to defects in the mitochondrial recycling machinery or an acceleration of mitochondrial production to compensate for the mitochondrial dysfunction. This gene encodes a synthase that produces 3’-phosphoadenosine-5’-phosphosulfate (PAPS), a key intermediate required for sulfation in eukaryotes [79]. Under normal circumstances, PPS-1 converts ATP into PAPS; a dysregulation in ATP production may influence other metabolic processes such as NADH oxidation, as evidenced by the low NADH levels in *pps-1(RNAi)* mutants. PPS-1 is a required gene in *C. elegans* and is the sole orthologue of two genes in humans, PAPSS1 and PAPSS2, that are both required and play nonredundant roles [79]. Mutations in PAPSS1 and PAPSS2 are associated with a broad range of phenotypes, including defects in bone and cartilage formation and hormone biosynthesis [80].

Although the function of PPS-1 is known, the connection between sulfation and mitochondria remains unclear, in part because of the variety of sulfated substrates. To obtain more information, we analyzed the genes connected to *pps-1*: *pop-1*, *gdph-2*, *sulp-3*, *sulp-5*, *cul-1*, and *dtmk-1*. This did not provide a clear answer either. One of the genes, *pop-1*, is involved in β-catenin binding, enzyme binding, and transcription corepressor binding, and is an orthologue for human LEF1 and TCF7L2. LEF1 is known to interact with SIRT1; overactivation of SIRT1 activates LEF1, driving increased mitochondrial biogenesis and reduced mitochondrial turnover, leading to increased ATP and ROS [81]. This is worth noting because there is a significant increase in ROS upon *pps-1* (*PAPSS1* orthologue) knockdown, and *pps-1* is predicted to be connected to *pop-1* (*LEF1* orthologue). This is similar to the increase in ROS driven by the SIRT1/LEF1 relationship. There is still little known about the relationship between PAPSS1 and SIRT1, or how SIRT1 affects mitochondrial function. This presents the possibility that our network can be used as a base to uncover novel protein interactions, such as the purported link between PAPSS1 and SIRT1 in mitochondrial maintenance.

Phenotypic assays of the genes in the panel also showed a significant increase in α-synuclein aggregates in a *C. elegans* neurodegenerative disease model. While this is not a faithful biomarker *per se*, misfolding and aggregation of α-synuclein in neural tissue is a hallmark of Parkinson’s disease [82]. Mitochondrial dysfunction is known to be implicated in PD, though there is still much that is unknown about what specific genes and pathways contribute to the progression of this disease [83,84,85,86]. Recent findings have indicated that downregulation of mitochondrial SIRT3 may play a role in the mitochondrial dysfunction in PD, and that α-synuclein may play a part in the decrease in SIRT3 expression through a decrease of phosphorylated AMPKα and phosphorylated CREB [85]. As shown above, the mitochondrial dysfunction caused by the RNAi we tested is very similar to the mitochondrial dysfunction observed within the disruption of genes within the sirtuin pathways (SIRT1 and SIRT3). While there is no known association between our validation gene panel and the aforementioned sirtuins, it may indicate possible links between them. Our gene set is proving to be a good reference for genes responsible not only for mitochondrial maintenance, but also for genes implicated in a variety of diseases.

In addition to measuring the phenotypic effects of knocking down the genes in our panel, we examined the gene set for gene connectivity and to determine whether any of the identified genes were plausible global regulators of mitochondrial maintenance. The most connected gene in our set was *rnr-2*, which encodes a subunit of ribonucleotide reductase, a cell cycle gene required for DNA synthesis, orthologous to human RRM2. Human RRM2 is known to be implicated in cancer and has functions that range from the regulation of BRCA1 to protection against ferroptosis [87,88]. While the literature connecting RRM2 and cancer is extensive, very little is known of the connection between RRM2 and mitochondrial maintenance. RRM2 is associated with a rare disease known as mitochondrial DNA depletion syndrome and is linked to mitochondrial DNA copy number in various models [89,90,91]. Mitochondrial DNA depletion syndrome is marked by malfunctions in mitochondrial DNA synthesis, reduced mitochondrial quantity, diverse clinical symptoms, and high infant mortality rates, possibly due to depletion of the nucleotide pools that RRM2 generates. Our data indicate that RRM2 is a hub for many of the genes in our set, potentially suggesting a larger role for RRM2 in mitochondrial maintenance than previously understood.

Overall, our findings provide insight into the genes and pathways that are needed for proper mitochondrial functions. Some of the genes in the set were previously not known to have roles in mitochondrial homeostasis. Out of the 139 genes from our set, 101 have no previously known relationship to mitochondrial function. This further exemplifies the need for a better understanding and discovery of genes related to mitochondrial function and overall cellular maintenance. Knockdown of multiple pathway members from the test panel disrupted mitochondrial function (e.g., fission–fusion imbalance, changes in mitochondrial mass, NADH, ROS, etc.) with concomitant phenotypic consequences (changes in pumping and/or brood size), including exacerbation of protein proteostatic defects. The immediate value of this gene set is in providing new fundamental knowledge about mitochondrial health. Additionally, this gene set may also be useful for assessing physiological manifestations of mitochondria-related disruption in diseases that may not be intuitively connected to mitochondrial dysfunction, such as proteostatic disorders or cancer.

## Figures and Tables

**Table 1:** A description of the eight-gene validation subset.

Gene in *C. elegans*	Protein	Human Orthologue	Direct Involvement with Mitochondrial Function?	Biological Function
*ogdh-1*	2-oxoglutarate dehydrogenase	OGDH	YES	Involved in the conversion of 2-oxoglutarate to succinyl-CoA
*kin-10*	Casein kinase II subunit beta	CSNK2B	NO	Modulates TRPP function [62], Involved in Wnt signaling [63]
*ego-2*	BRO1 domain-containing protein	PTPN23	NO	Promotes Notch signaling [64]
*bar-1*	Beta-catenin armadillo-related protein 1	CTNNB1	NO	Regulates Hox gene *lin-39* expression through Wnt signaling [65]
*pps-1*	3′-phosphoadenosine-5′-phosphosulfate synthase	PAPSS1	YES	Involved in sulfate metabolism [66]
*exos-9*	Exosome complex component RRP45	EXOSC9	NO	Not well studied but exhibits exonuclease activity
*cdc-37*	Probable Hsp90 co-chaperone	CDC37	NO *	Cofactor for many kinases and is involved in ATPase activity [67]
*cul-1*	Cullin-1	CUL1	NO	Involved in protein degradation [68]

* While direct interaction of CDC37 with mitochondria has not been reported, its probable co-chaperone HSP90 has. Note: Eight genes were chosen to represent the gene set as a whole. Seven out of the eight genes were in separate clusters in a *k*-means clustering analysis (Figure S1). These included genes with both known and presumed (on the basis of this set) functions in mitochondrial health maintenance.

**Table 2:** Summary table of mitochondrial phenotypes.

Gene in *C. elegans*	NADH	ROS	Mitochondrial Mass	Membrane Potential	Oxygen Consumption
*ogdh-1*	**NS**	**NS**	**NS**	**0.40X**	**0.53X**
*kin-10*	**NS**	**NS**	**NS**	**0.57X**	**1.65X**
*ego-2*	**0.56X**	**2.57X**	**2.49X**	**NS**	**0.24X**
*bar-1*	**0.48X**	**1.92X**	**2.40X**	**NS**	**0.39X**
*pps-1*	**0.46X**	**3.12X**	**3.12X**	**NS**	**0.64X**
*exos-9*	**NS**	**NS**	**1.65X**	**0.29X**	**0.69X**
*cdc-37*	**NS**	**NS**	**NS**	**NS**	**NS**
*cul-1*	**NS**	**NS**	**NS**	**0.51X**	**NS**

Note: Mitochondrial fragmentation and mitochondrial surveillance activation data were not included in this table. Red denotes a significant increase. Blue denotes a significant decrease.

## Data Availability

Strains are available upon request. The authors affirm that all data necessary for confirming the conclusions of the article are present within the article, figures, and tables.

## Supplementary Materials

The following supporting information can be downloaded at: https://www.mdpi.com/article/10.3390/genes14051009/s1, Figure S1. A K-means clustering analysis is used to choose genes in the validation panel. A K-means clustering analysis separated the gene set into eight clusters, and seven out of the eight genes in the validation panel were present in different clusters. Figure S2. Mitochondrial functional assay data without normalization to mitochondrial mass. (A) Bar graph of steady-state ratio of NADH–NAD+ using a conformation-dependent fluorometric reporter. Young adult worms carrying the reporter were reared on the indicated RNAi constructs. (B) Bar graph of steady-state reactive oxygen species, as measured by conversion of dihydroethidium to a fluorescent state in young adult worms reared on RNAi targeting the indicated gene. Fluorescence was measured using a Cytation5 Cell Imaging Multi-Mode Reader. (C) Bar graph of mitochondrial membrane potential, as measured by the dye MitoTracker Red CMXRos in young adult worms reared on RNAi targeting the indicated gene. Fluorescence was measured using flow vermimetry. (D) Bar graph of basal oxygen consumption in young adult worms reared on RNAi targeting the indicated gene, as measured by a Clark electrode. Statistical significance in all panels was calculated using one-way ANOVA analysis followed by a Dunnett’s post hoc test. * *p* < 0.05, ** *p* < 0.01, *** *p* < 0.001. * indicates significant increase compared to *EV*, * indicates significant decrease compared to *EV*. Figure S3. HyPer reporter as a measure of hydrogen peroxide. Bar graph of steady-state hydrogen peroxide levels using a fluorometric reporter. Young adult worms carrying the reporter were reared on the indicated RNAi constructs. Statistical significance in all panels was calculated using one-way ANOVA analysis followed by a Dunnett’s post hoc test. *** *p* < 0.001. * indicates significant increase compared to *EV*. Figure S4. Nonmitochondrial surveillance reporters measured effects of RNAi on overall cellular health. (A) Bar graph of *gst-4*::GFP oxidative stress reporter signal in young adult worms reared on RNAi targeting the indicated gene. Juglone was used to induce the empty vector positive control. (B) Bar graph of *hsp-4*::GFP UPR^ER^ reporter signal in young adult worms reared on RNAi targeting the indicated gene. Tunicamycin was used to induce the empty vector positive control. (C) Bar graph of *rpt-3*::GFP proteasome stress reporter signal in young adult worms reared on RNAi targeting the indicated gene. Bortezomib was used to induce the empty vector positive control. Statistical significance in all panels was calculated using one-way ANOVA analysis followed by a Dunnett’s post hoc test. *** *p* < 0.001. * indicates significant increase compared to *EV*. Figure S5. N2 and *fer-15;fem-1* mutant effect on fecundity. (A) Bar graph of worm fecundity in adult N2 worms reared on RNAi targeting the indicated gene. (B) Bar graph of *fer-15;fem-1* worm fecundity in adult worms reared on RNAi targeting the indicated gene. Statistical significance in all panels was calculated using one-way ANOVA analysis followed by a Dunnett’s post hoc test. *** *p* < 0.001. * indicates significant decrease compared to *EV*. Table S1. List of genes that passed the PINK-1 screen. This list contains all the genes that, when knocked down, exhibited a significant increase in fluorescence compared to the empty vector control in the PINK-1 screen. Table S2. List of genes that passed the CCCP screen. This list contains all the genes that, when knocked down, exhibited a significant increase in fluorescence compared to the empty vector control in the CCCP screen. Table S3. List of seed genes. This list contains the genes that were left after passing through all the screens, and were used as a seed list to obtain more genes to screen. Table S4. List of ~400 *C. elegans* genes with a significant connection to seed set. Approximately 400 genes that are highly connected to the initial gene set were obtained using WormNetV3. Connections are considered significant when connectivity *p*-values calculated by WormNetV3 were < 0.05; Table S5. List of human orthologs for 139 *C. elegans* genes of expended set. Orthologs were identified using OrthoList, DIOPT, and Wormbase, and manually curated. Table S6. List of enriched OMIM categories. OMIM disease enrichment shows a wide variety of diseases that are enriched in the mitochondrial health gene set. Statistical significance (*p*-values) and false discovery rate (*q* values) are shown.
