# Supplementary material for: Examining Sporadic Cancer Mutations Uncovers a Set of Genes Involved in Mitochondrial Maintenance
Source: Genes (Basel). 2023 Apr 29;14(5):1009. doi: 10.3390/genes14051009 (PMC10218105; doi:10.3390/genes14051009)
Supplement: Supplementary file 1 [file genes-14-01009-s001.zip › Supplemental Figures Compiled.pdf]

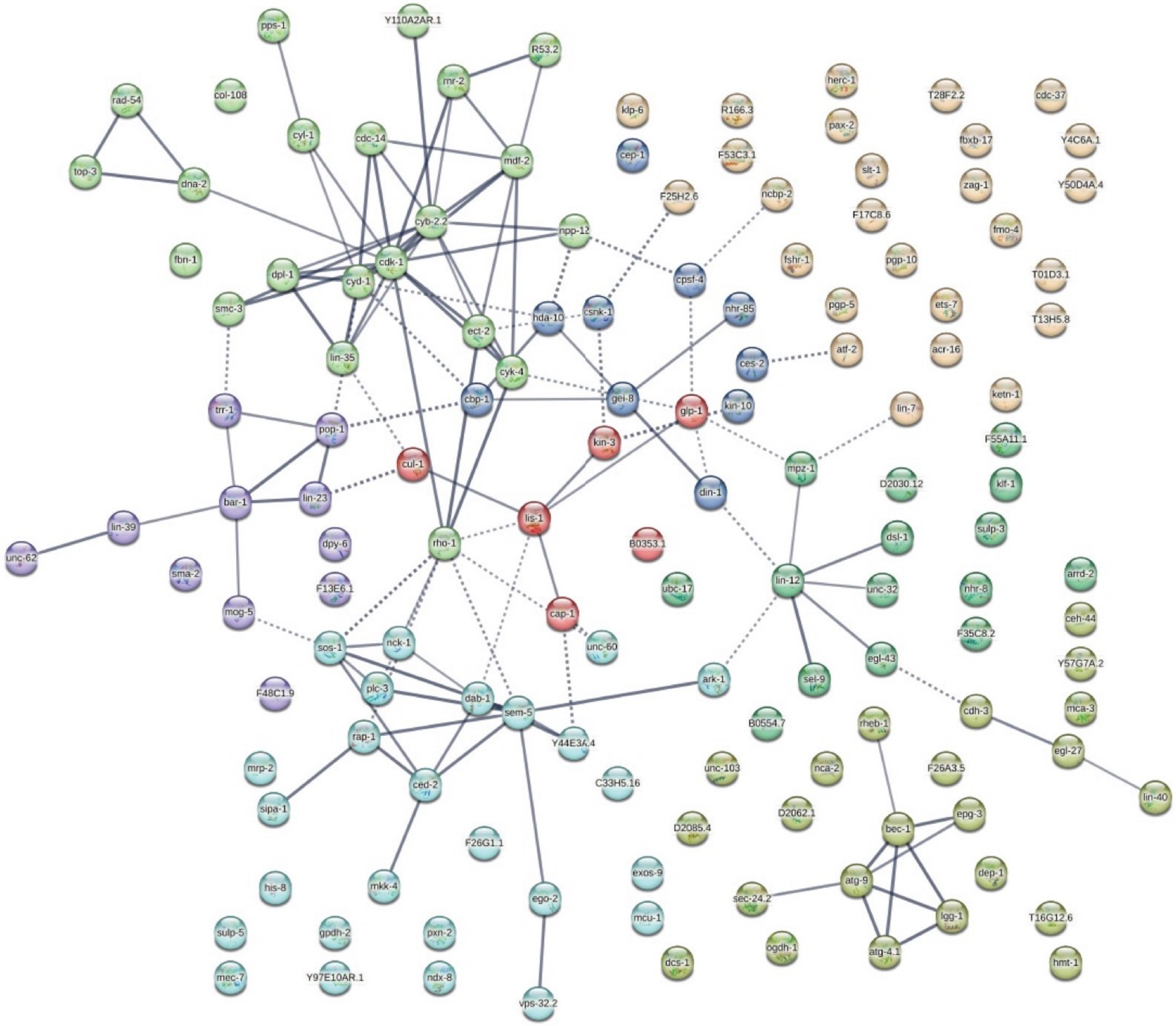

Supplemental Figure S1

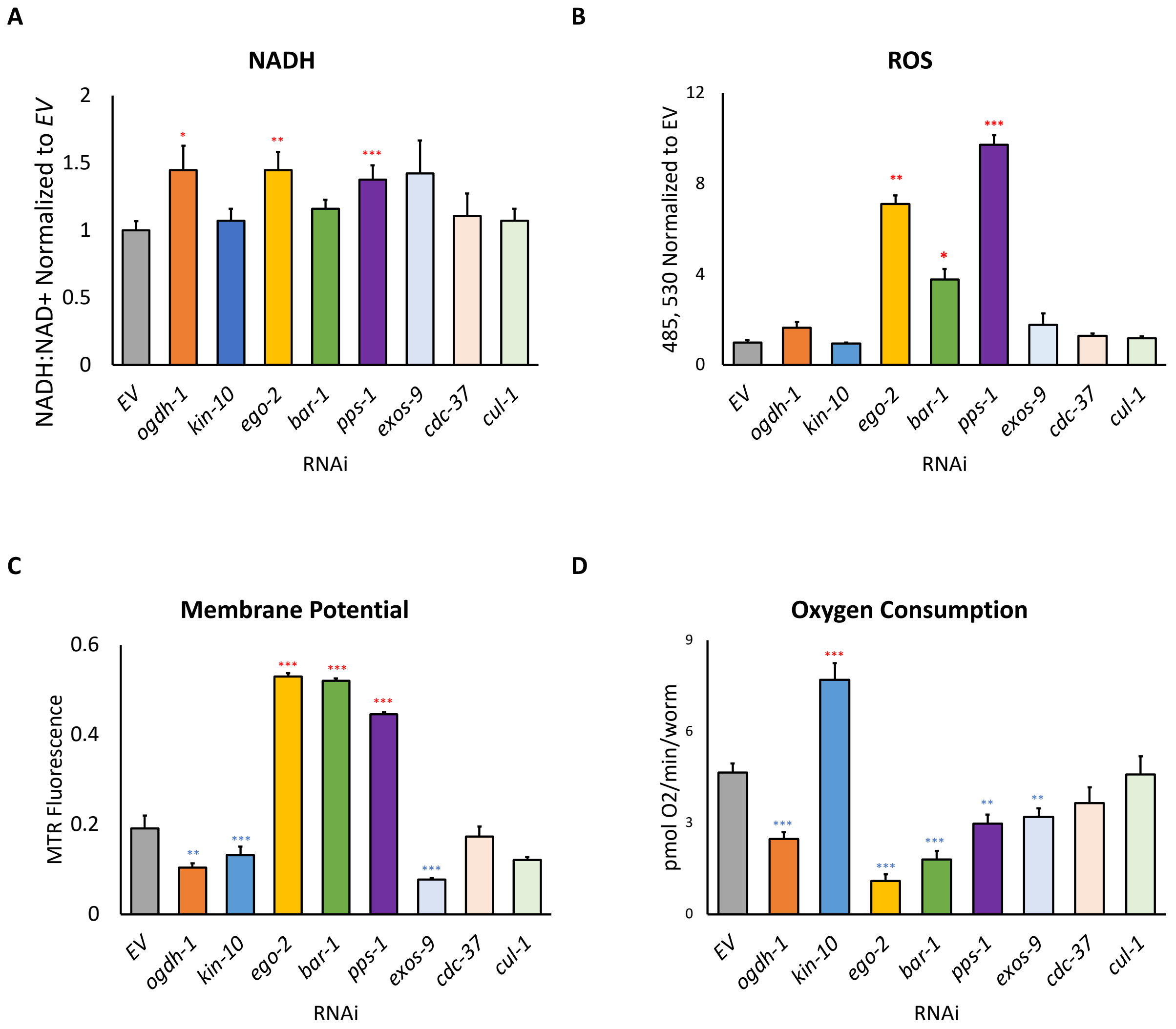

Supplemental Figure S2

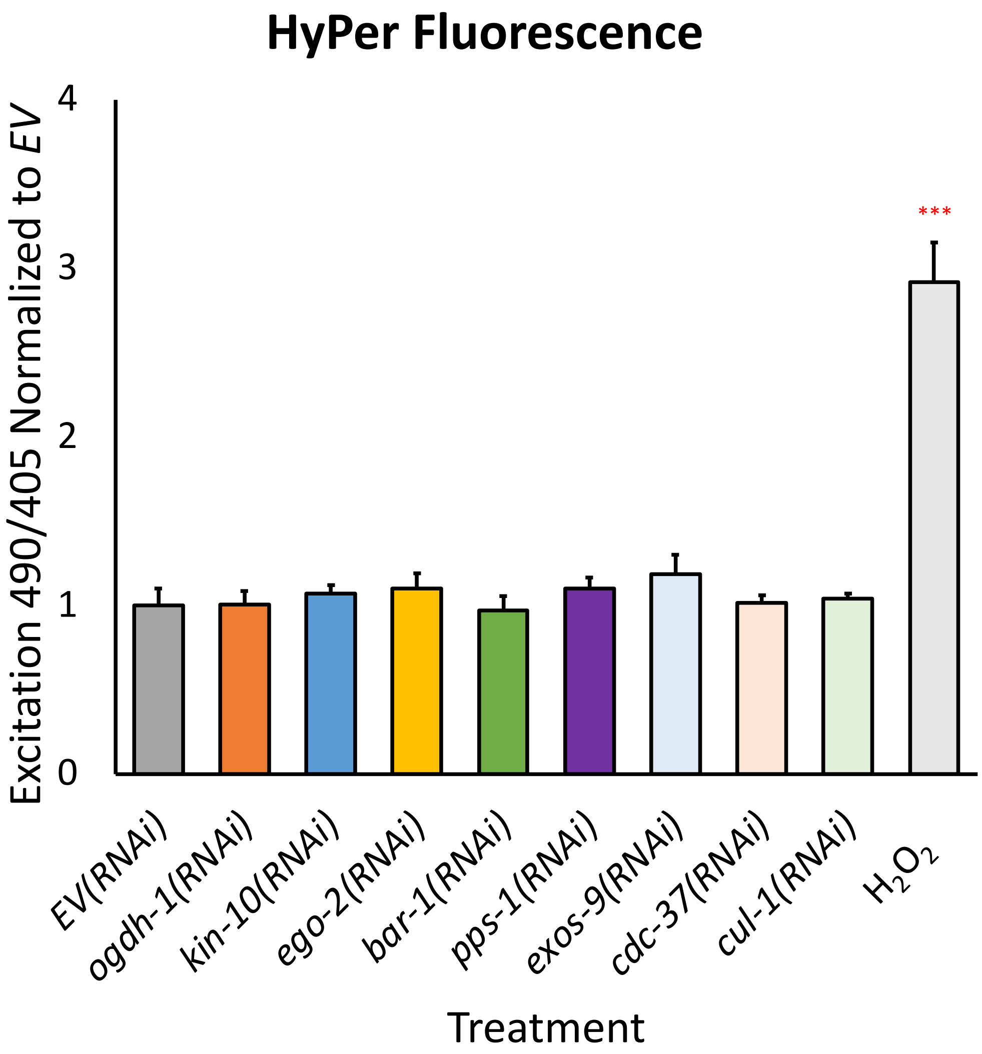

Supplemental Figure S3

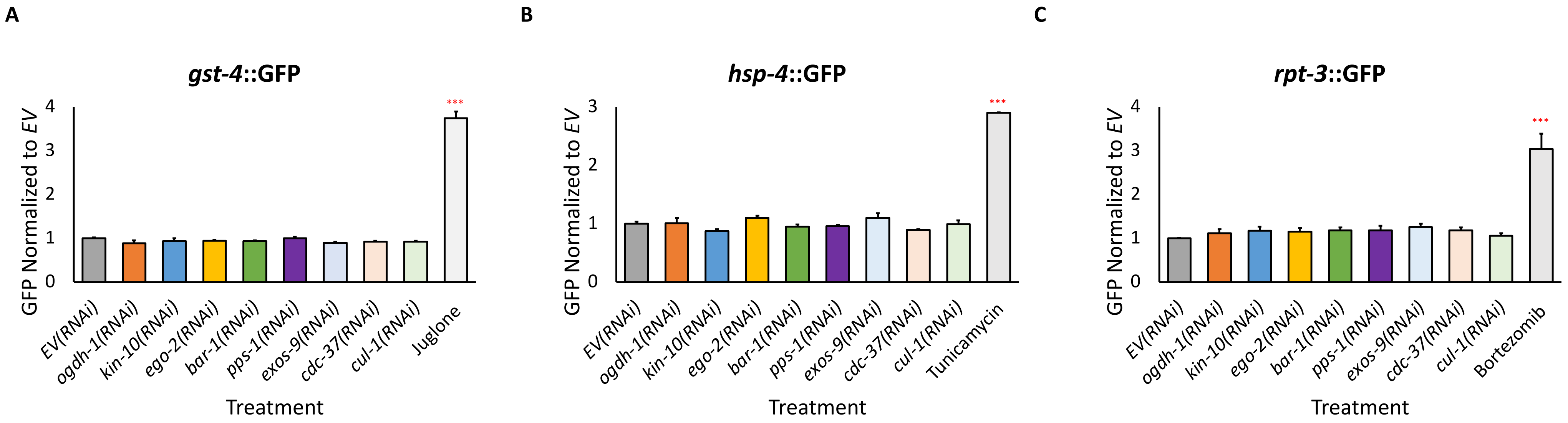

Supplemental Figure S4

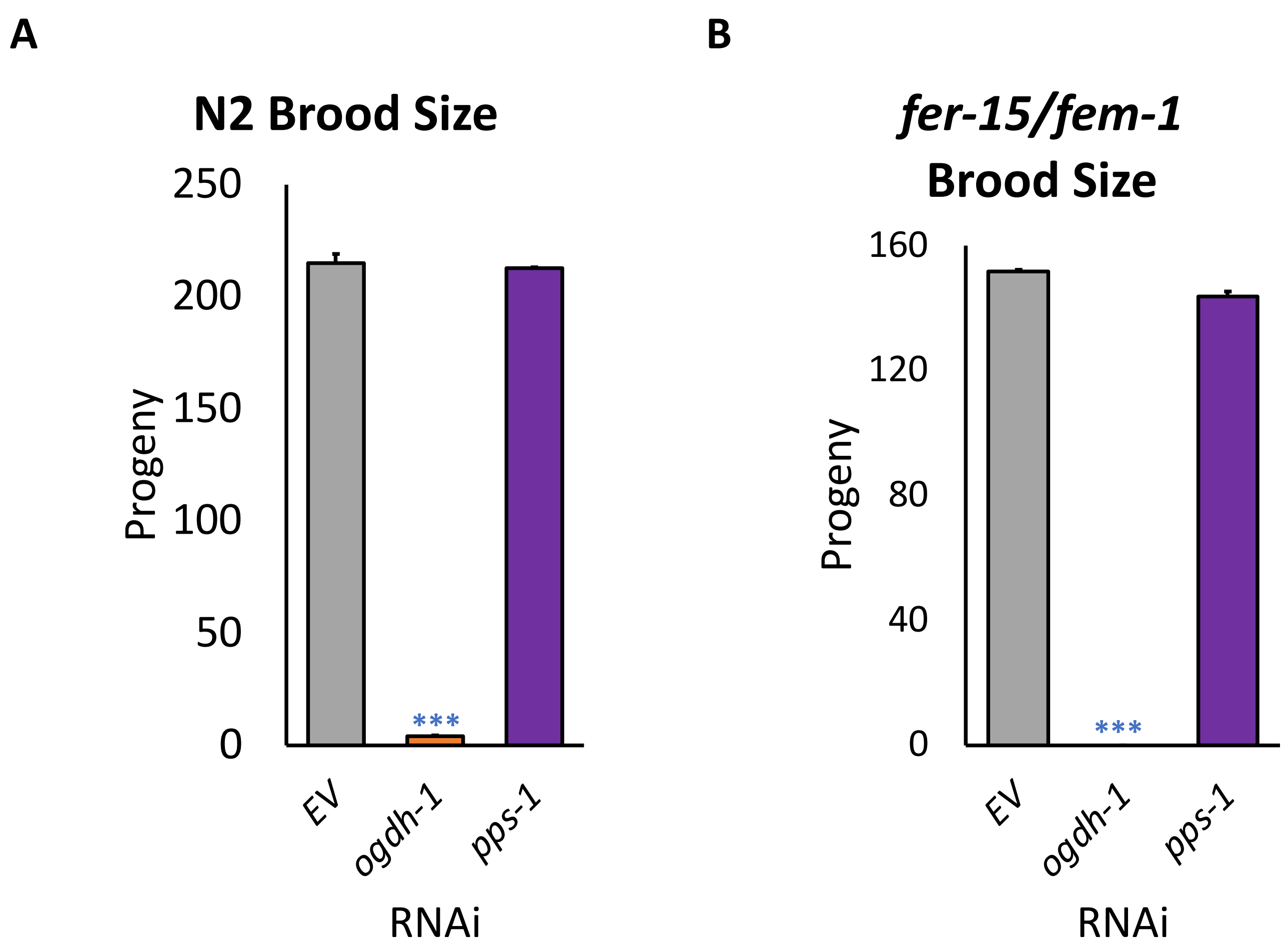

Supplemental Figure S5
